# Exploring the molecular mechanism of ginseng against anthracycline-induced cardiotoxicity based on network pharmacology, molecular docking and molecular dynamics simulation

**DOI:** 10.1186/s41065-024-00334-y

**Published:** 2024-09-06

**Authors:** Lin Xie, Hanze Liu, Ke Zhang, Yijun Pan, Mengyao Chen, Xiangyue Xue, Guoxing Wan

**Affiliations:** 1 Department of Oncology, Institute of Medicine and Nursing, Renmin Hospital, Hubei University of Medicine, Shiyan, Hubei 442000 China; 2 Department of Oncology, Renmin Hospital, Hubei University of Medicine, 39 Chaoyang Road, Shiyan, Hubei 442000 China

**Keywords:** Ginseng, Anthracycline-induced cardiotoxicity, Network pharmacology, Molecular docking

## Abstract

**Background:**

Previous clinical and basic studies have revealed that ginseng might have cardioprotective properties against anthracycline-induced cardiotoxicity (AIC). However, the underlying mechanism of ginseng action against AIC remains insufficiently understood. The aim of this study was to explore the related targets and pathways of ginseng against AIC using network pharmacology, molecular docking, cellular thermal shift assay (CETSA) and molecular dynamics (MD) simulations.

**Results:**

Fourteen drug-disease common targets were identified. Enrichment analysis showed that the AGE-RAGE in diabetic complications, fluid shear stress and atherosclerosis, and TNF signaling pathway were potentially involved in the action of ginseng against AIC. Molecular docking demonstrated that the core components including Kaempferol, beta-Sitosterol, and Fumarine had notable binding activity with the three core targets CCNA2, STAT1, and ICAM1. Furthermore, the stable complex of STAT1 and Kaempferol with favorable affinity was further confirmed by CETSA and MD simulation.

**Conclusions:**

This study suggested that ginseng might exert their protective effects against AIC through the derived effector compounds beta-Sitosterol, Kaempferol and Fumarine by targeting CCNA2, STAT1, and ICAM1, and modulating AGE-RAGE in diabetic complications, fluid shear stress and atherosclerosis, and TNF signaling pathways.

**Supplementary Information:**

The online version contains supplementary material available at 10.1186/s41065-024-00334-y.

## Introduction

Adriamycin, also known as doxorubicin (DOX), is an anthracycline-based chemotherapeutic agent that exhibits efficacy in treating various types of solid tumors and hematologic malignancies, including osteosarcoma, malignant lymphoma, and breast cancer [1]. However, its use is associated with a dose-dependent risk of cardiotoxicity, which can lead to severe consequences such as progressive heart failure and irreversible cardiac insufficiency [2]. Studies have reported a graded increase in the likelihood of cardiotoxicity occurring at cumulative doses of 400, 500, and 550 mg/m^2^ with the use of DOX, corresponding to probabilities of approximately 5%, 16%, and 26%, respectively [3]. The pathophysiological mechanisms underlying adriamycin-induced cardiotoxicity (AIC) remain incompletely understood, although oxidative stress induced by DOX is widely believed to play a crucial role in this process [4]. It has been postulated that DOX triggers the production of excessive reactive oxygen species (ROS), leading to lipid peroxidation of cell membranes and damage to the cellular mitochondrial structure, ultimately resulting in the damage and death of cardiomyocytes [5]. Unfortunately, dexrazoxane (DEX), the sole clinically approved agent for preventing AIC, is hampered by its possible interference on DOX efficacy and risk of secondary malignancy, limiting its widespread clinical utility [6]. Consequently, there exists a pressing need for the development of novel therapeutics capable of mitigating AIC.

Ginseng, a plant commonly utilized in traditional Chinese medicine, has been recognized for its cardioprotective properties and incorporated into various classical Chinese medical formulations as the "monarch drug". Previous studies demonstrated that ginseng exhibited a range of pharmacological activities, including the inhibition of cardiomyocyte apoptosis and mitochondrial swelling, reduction of oxidative stress-induced cellular damage, and notable efficacy in treating cardiovascular diseases such as angina pectoris, coronary artery atherosclerosis, and myocardial ischemia–reperfusion injury [7]. Previous animal studies and clinical trials have shown that ginseng could alleviate adriamycin-induced cardiac injury. In a randomized, double-blind, placebo-controlled clinical trial, Hamidian et al. demonstrated the protective effect of ginseng supplementation against doxorubicin-induced early cancer therapeutics-related cardiac dysfunction and early decline in left ventricular ejection fraction in breast cancer patients [8]. Basic research conducted by You and colleagues suggested that ginseng might play a role in alleviating adriamycin-induced heart failure in rodents [9]. In previous pharmacological studies, ginseng glycoproteins and ginsenosides derived from ginseng were demonstrated to exhibit the cardio-protective effect through anti-inflammatory, antioxidant and anti-apoptotic effects [10–12]. Nevertheless, further research is necessary to fully understand the mechanisms behind ginseng's cardioprotective effects, particularly given its complex composition and multifaceted targets.

The benefits of network pharmacology reside in its comprehensive and integrated approach to explore the therapeutic potential of drugs on diseases, which is achieved through the construction of drug-disease-target-constituent networks. This method aligns with the holistic perspective of traditional Chinese medicine, which emphasizes the interconnectedness of multiple components, pathways, and targets [13].

**Fig. 1:**
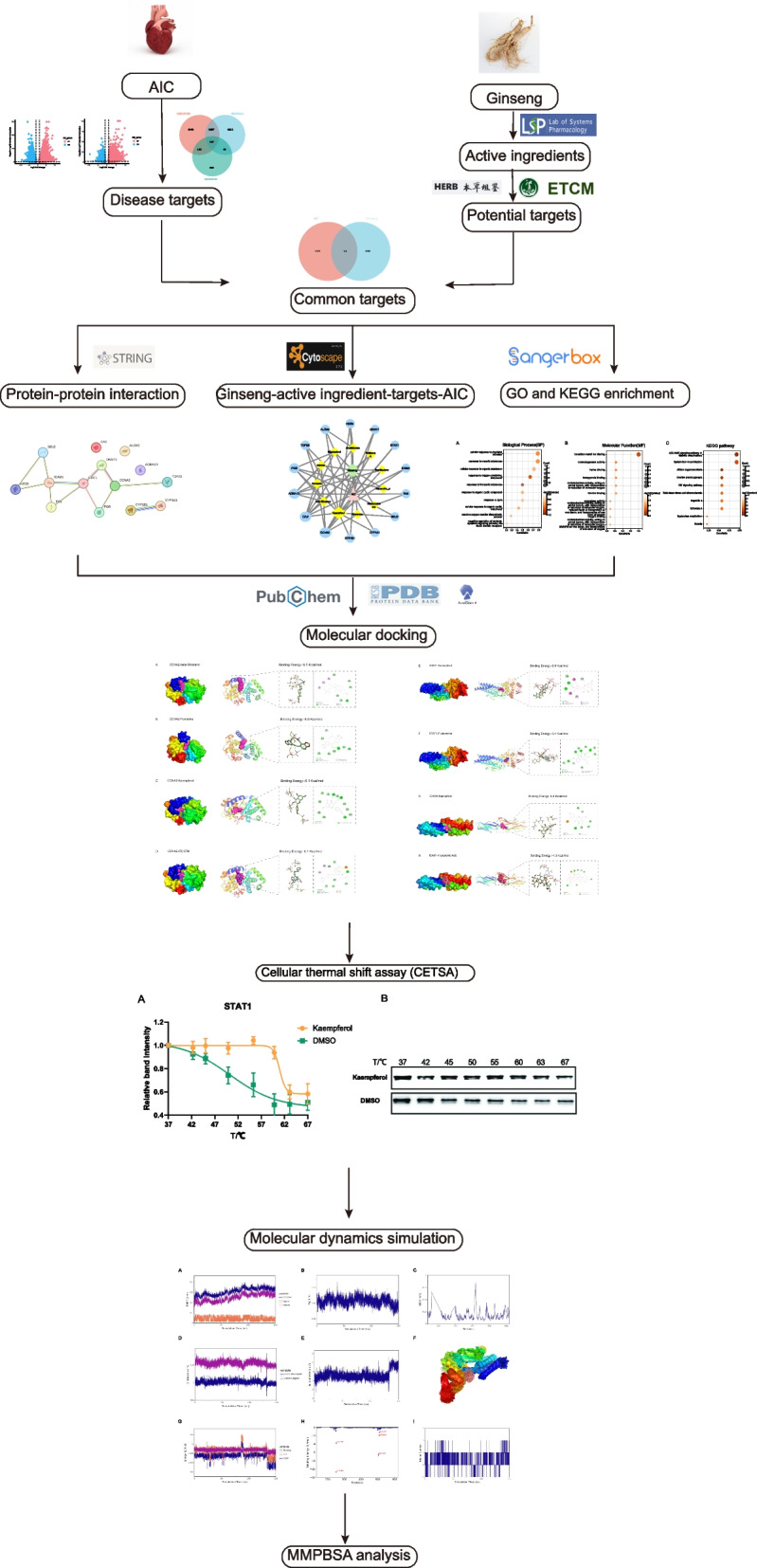
The workflow to investigate the molecular mechanism of ginseng in the treatment of AIC

Hence, we employed network pharmacology, molecular docking, cellular thermal shift assay (CETSA) and molecular dynamics (MD) simulation techniques to investigate the potential therapeutic targets and mechanisms of ginseng in mitigating AIC. Our findings may provide a foundation for future drug development endeavors targeting AIC. The workflow chart was shown in Fig. 1.

## Materials and methods

### Identification of active ingredients and targets of ginseng

To identify the active ingredients of ginseng, the TCMSP database (https://tcmspw.com/tcmsp.php) was queried using the keyword "Ginseng". The employed screening criteria were an oral bioavailability (OB) of ≥ 30% and a drug-like activity (DL) of ≥ 0.18. The effective targets of ginseng were obtained from three databases, namely TCMSP, HERB (http://herb.ac.cn/), and ETCM (http://www.tcmip.cn/ETCM/). To standardize the names of the screened target proteins obtained from TCMSP, the Uiprot (https://www.uniprot.org/) and GeneCards (https://www.genecards.org/) databases were utilized to obtain their respective standard gene names.

### Identification of AIC targets

The present investigation utilized two transcriptomic datasets, specifically GSE157282 and GSE76314 [14, 15], which were derived from human induced pluripotent stem cell-induced cardiomyocytes (hiPSC-CMs) focusing on AIC. The GSE157282 dataset was comprised of four groups (control, doxorubicin, R-2HG and R-2HG plus doxorubicin treatment). Of which, the control and doxorubicin groups were chosen for differentially expressed gene analysis. While, samples in GSE76314 were derived from breast cancer patients who were suffered from clinical AIC or not (three patients per group). The hiPSC-CMs from each group were subjected to doxorubicin treatment (0μM or 1μM). The data involving hiPSC-CMs derived from patients with clinical AIC were chosen, and the samples subjected to doxorubicin with 1 μM were set as case group while 0μM as control group. Following retrieval of the raw read count data from the Gene Expression Omnibus (GEO) database, we employed the "DEseq2" package to perform differential gene analysis. Subsequently, the resulting data were visualized via a volcano plot created with ImageGP (http://www.bic.ac.cn/BIC/). The differentially expressed genes (DEGs) for GSE15728 and GSE76314 were determined with the criteria of |LogFC|> 1 and padj < 0.05. Furthermore, additional AIC targets were obtained by searching GeneCards database using the terms of “adriamycin cardiotoxicity” OR “doxorubicin cardiotoxicity” OR “adriamycin heart failure” OR “doxorubicin cardiomyopathy”. Subsequently, the intersection of these DEGs from GSE157282, GSE76314 and GeneCards were performed to result in AIC targets.

### Screening of potential targets and core active ingredients of ginseng against AIC and construction of ginseng-AIC-ingredients-targets network

The shared genes between the AIC and ginseng targets were considered the potential target genes of ginseng against AIC, which was visualized with a venn diagram using the online tool SangerBox (http://vip.sangerbox.com). Using Cytoscape 3.7.2 software (http://www.cytoscape.org/), the ginseng-active ingredients-targets-AIC network was then constructed. The top three active ingredients with the highest number of targets in the network were considered core active ingredients.

### Protein–protein interaction network construction, topology analysis and core target screening

The shared targets of ginseng and AIC were utilized as input for the STRING database (https://string-db.org/) for protein–protein interaction (PPI) analysis. The species of choice was homo sapiens and TSV files containing PPI network was obtained. This file was subsequently imported into Cytoscape 3.7.2 for further analysis. Network topology analysis was conducted employing the CytoNCA plugin, encompassing parameters such as DC, BC, CC, EC, LAC, and NC. A target was deemed a core target if all six parameter values were greater than or equal to the median value of their respective parameter groups, warranting its inclusion in subsequent analysis.

### Gene Ontology (GO) and Kyoto Encyclopedia of Genes and Genomes (KEGG) pathway enrichment analysis

GO and KEGG enrichment analysis with “clusterProfiler” package [16] were performed to explore possible biological process (BP), molecular function (MF) and signaling pathway involved in ginseng action on AIC. The results of GO and KEGG results were visualized using SangerBox.

### Molecular docking

Molecular docking was a computational approach to predict the docked poses and binding affinities between the proteins and ligands. Before performing the molecular docking process, the re-docking of co-crystal ligand (if available) was performed to assess the performance of the employed docking technique. The root mean square deviation (RMSD) of the re-docked conformation was compared to the original experimentally validated co-crystal conformation, and a RMSD ≤ 2 Å indicated acceptable performance of the employed docking technique.

To perform molecular docking, the 2D structures of active ingredients were downloaded from the PubChem database (https://pubchem.ncbi.nlm.nih.gov/). Then compounds were energy-minimized using Chem3D and stored in mol2 format. The protein crystal structures of the core targets CCNA2 (PDB ID: 1FIN), STAT1 (PDB ID: 1BF5), and ICAM1 (PDB ID: 1IAM) were obtained from the PDB database (http://www.rcsb.org/). Water molecules and original ligands were removed from the core target proteins utilizing PYMOL software, and missing protein structures were repaired by SPDBV software. Subsequently, the protonation status of the titrable amino acids and ligand were investigated. Next, both receptor proteins and chosen compounds were saved in PDBQT files after adding hydrogen atoms, protonating, and calculating charge using Autodock. The location of binding sites for molecular docking was optimized by modifying the central coordinate and size of the docking protein. Finally, Autodock vina was used to dock the core receptor proteins with small molecule compound ligands. Each molecular docking generated ten conformations and the best docking conformation according to the binding energy and site were selected as the ultimate docking result. Of which, a binding energy threshold of less than − 5.0 kcal/mol was employed as the screening criterion, with lower binding energies indicating a stronger affinity between the key targets and core active ingredients. Subsequently, through PYMOL and Discovery Studio, the interactions of compounds and proteins were visualized. Moreover, the docking results were compared to a reference drug/inhibitor to assess the reliability. In this study, (2R)-2-({9-(1-methylethyl)-6-[(4-pyridin-2-ylbenzyl)amino]-9H-purin-2-yl}amino)butan-1-ol [abbreviated as (R)-CR8], Fludarabine, and Hyaluronic acid, as inhibitors of CCNA2, STAT1, and ICAM1 proteins, respectively, were used as reference drug/inhibitor for molecular docking.

### Cellular Thermal Shift Assay (CETSA)

The binding ability of Kaempferol to STAT1 was examined by CETSA. H9C2 cells, purchased from Zhongqiaoxinzhou Biotechnology Co., Ltd. (ZQ0102, Shanghai, China), were routinely cultured in DMEM supplemented with 10% fetal bovine serum and 1% penicillin–streptomycin antibiotics. All cells were incubated at 37 °C in a humidified incubator with 5% CO2. H9C2 cells were treated with 20 μM Kaempferol for 24h, while the control group was treated with the equivalent volume of DMSO. Two groups of cells were collected and resuspended in PBS containing protease inhibitor, separately. Subsequently, samples were divided into 8 equal parts, and each part was heated individually at designated temperatures (37–67 ℃) for 3 min via the PCR instrument. Afterwards, the lysates were cooled at room temperature by 3 min. Next, samples were repeatedly freeze-thawed 3 times by using liquid nitrogen. Following centrifugation at 20,000 g for 20 min at 4℃, the supernatants were extracted, and the expression of STAT1 was analyzed by Western blot. Primary antibodies STAT1 and secondary antibodies were purchased from Proteintech Group, Inc (Wuhan, China). Kaempferol were purchased from MedChemExpress (Shanghai, China).

### Molecular dynamics simulation

Molecular dynamics (MD) simulation was further performed to investigate the structural stability of the protein–ligand complexes using GROMACS 2022 software. To meet the high-efficiency of MD, a truncated construct of the protein which had no influence on the docking of protein–ligand was used if necessary [17]. Protein topology file was generated by AMBER14SB force field, and ligand topology file was obtained by GAFF force field using Sobtop software (http://sobereva.com/soft/Sobtop/). Multiwfn software was used to calculate the restrained electrostatic potential (RESP) charges of ligands [18]. Docked complexes were solvated in a cubic water box with 10 Å periodic boundary from the protein border by applying TIP3P water model. CL-and NA + ions were added to the system to maintain electrical neutrality of the environment and energy of complexes were minimized in 5000 steps by using steepest descent method. During the MD simulations, the Linear Constraint Solver (LINCS) algorithm was used to constrain the lengths of hydrogen bonds [19], and the Particle Mesh Ewald (PME) method was applied for the calculation of electrostatic interactions [20]. Meanwhile, the Berendsen weak coupling method was implemented during the NVT equilibration stage to maintain a consistent temperature of 300 K [21]. To equilibrate the entire system, NVT ensemble for 10 ns at 300 k and NPT ensemble 10 ns at 1 bar were employed. Finally, MD simulations of the docking complex was carried out for 150 ns in 300 K temperature and 1 bar pressure with a time step of 2 femtoseconds. Following the simulation completed, the simulation trajectories were used to generated root mean square deviation (RMSD), root mean square fluctuation (RMSF), radius of gyration (Rg), buried solvent accessible surface area (Buried SASA), number of hydrogen bonds, binding conformational superposition and so on.

### Binding free energy calculation (MM/PBSA)

Following the completion of the MD simulation, 1000 frames were extracted from the last 10 ns of the simulation trajectories to calculate the binding free energy using the Molecular Mechanics Poisson-Boltzmann Surface Area (MMPBSA) method. Of note, entropy change was not considered with the consideration of high consumption of computational resources and low accuracy. The calculation formula was as follows:

$$\Delta {\text {Gbind}}=\Delta {\text {Gcomplex}}-\left(\Delta {\text {Greceptor}}+\Delta {\text {Gligand}}\right)$$

where ΔGbind represented the total binding free energy of the protein–ligand complex, while ΔGreceptor and ΔGligand were the binding free energy of protein and ligand, respectively.

## Results

### Active ingredients and corresponding targets identification of ginseng

Based on the OB and DL values, 22 active ingredients of ginseng were identified with the TCMSP database (Table 1). The corresponding targets of these 22 active ingredients were obtained through the TCMSP, HERB, and ETCM databases, resulting in a total of 281 target genes (Supplementary Table S1).

**Table 1:** Characteristics of active ingredients in Ginseng

Mol ID	Ingredient	OB(%)	DL
MOL002879	Diop	43.59	0.39
MOL000449	Stigmasterol	43.83	0.76
MOL000358	beta-Sitosterol	36.91	0.75
MOL003648	Inermin	65.83	0.54
MOL000422	Kaempferol	41.88	0.24
MOL004492	Chrysanthemaxanthin	38.72	0.58
MOL005308	Aposiopolamine	66.65	0.22
MOL005314	Celabenzine	101.88	0.49
MOL005317	Deoxyharringtonine	39.27	0.81
MOL005318	Dianthramine	40.45	0.2
MOL005320	Arachidonate	45.57	0.2
MOL005321	Frutinone A	65.9	0.34
MOL005344	Ginsenoside rh2	36.32	0.56
MOL005348	Ginsenoside-Rh4_qt	31.11	0.78
MOL005356	Girinimbin	61.22	0.31
MOL005357	Gomisin B	31.99	0.83
MOL005360	Malkangunin	57.71	0.63
MOL005376	Panaxadiol	33.09	0.79
MOL005384	Suchilactone	57.52	0.56
MOL005399	Alexandrin_qt	36.91	0.75
MOL005401	Ginsenoside Rg5_qt	39.56	0.79
MOL000787	Fumarine	59.26	0.83

*OB* oral bioavailability, *DL* drug likeness

### Identification of target genes related to AIC

**Fig. 2:**
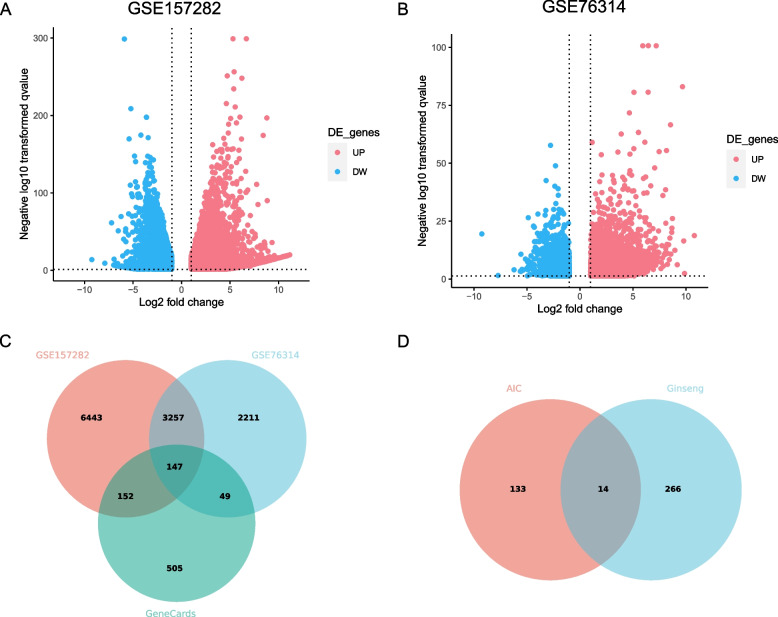
Plots of ginseng and AIC targets. **A** Volcano diagram of DEGs for GSE157282. **B** Volcano diagram of DEGs for GSE76314. **C** Venn diagram of AIC targets. **D** Venn diagram of common targets between ginseng and AIC

According to the differential gene analysis, a total of 10,047 DEGs from the GSE157282 dataset and 5,664 DEGs from the GSE76314 dataset were identified, respectively, which were presented as the volcano plots (Fig. 2A, 2B). The intersection analysis yielded a total of 3,404 DEGs. Finally, a total of 147 overlapping targets were identified by intersecting 3404 DEGs and 853 targets extracted from the GeneCards database, which were considered target genes related to AIC (Fig. 2C).

### Network construction of ginseng-ingredients-targets-aic and identification of core active ingredients and targets of ginseng against AIC

**Fig. 3:**
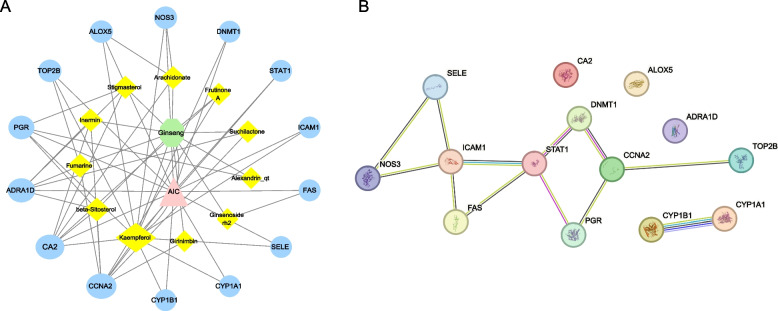
Plots of ginseng-ingredients-targets-AIC and protein–protein interaction network. **A** Ginseng-ingredients-targets-AIC relationship network (green octagon represents ginseng, pink triangle represents AIC, yellow rhombus represents ginseng active ingredients, blue circles represent potential targets, and gray connecting lines represent interactions among the nodes). **B** Protein–protein interaction (PPI) network of ginseng targets in the treatment of AIC

By taking the intersection of the target genes for the active ingredients of ginseng and the target genes for AIC, a total of 14 shared targets were identified (Fig. 2D). The active ingredients and shared targets were further used to construct the ginseng-ingredients-targets-AIC network with Cytoscape software (Fig. 3A), utilizing the protein–protein interactions obtained from the STRING database (Fig. 3B). According to the established criterion, topological network analysis identified three core active ingredients, including Kaempferol, beta-Sitosterol, and Fumarine, along with three core targets (CCNA2, STAT1, and ICAM1).

### GO and KEGG enrichment analysis

**Fig. 4:**
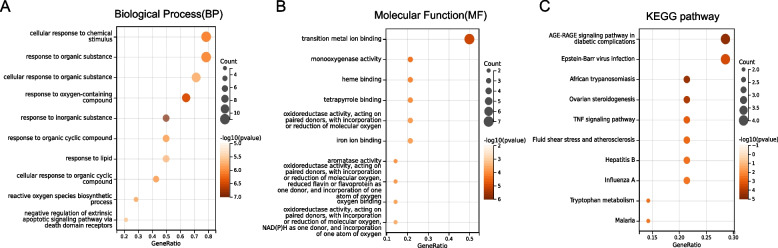
GO and KEGG pathway enrichment analysis of common targets. **A** GO in terms of biological processes. **B** GO in terms of molecular function. **C** KEGG pathway analysis

The 14 shared genes were subjected to GO and KEGG enrichment analysis using SangerBox based on clusterProfiler package, with a threshold of *p* < 0.05. BP term results revealed that cellular response to chemical stimulus, response to organic substance along with others may be involved in ginseng action. MF term results revealed the involvement of monooxygenase activity, tetrapyrrole binding, and oxidoreductase activity along with others in ginseng action. The KEGG enrichment analysis identified 17 signaling pathways, which mainly involved AGE-RAGE in diabetic complications, fluid shear stress and atherosclerosis, and TNF signaling pathway, etc. The top 10 results from each category were presented as Fig. 4.

### Molecular docking results

**Fig. 5:**
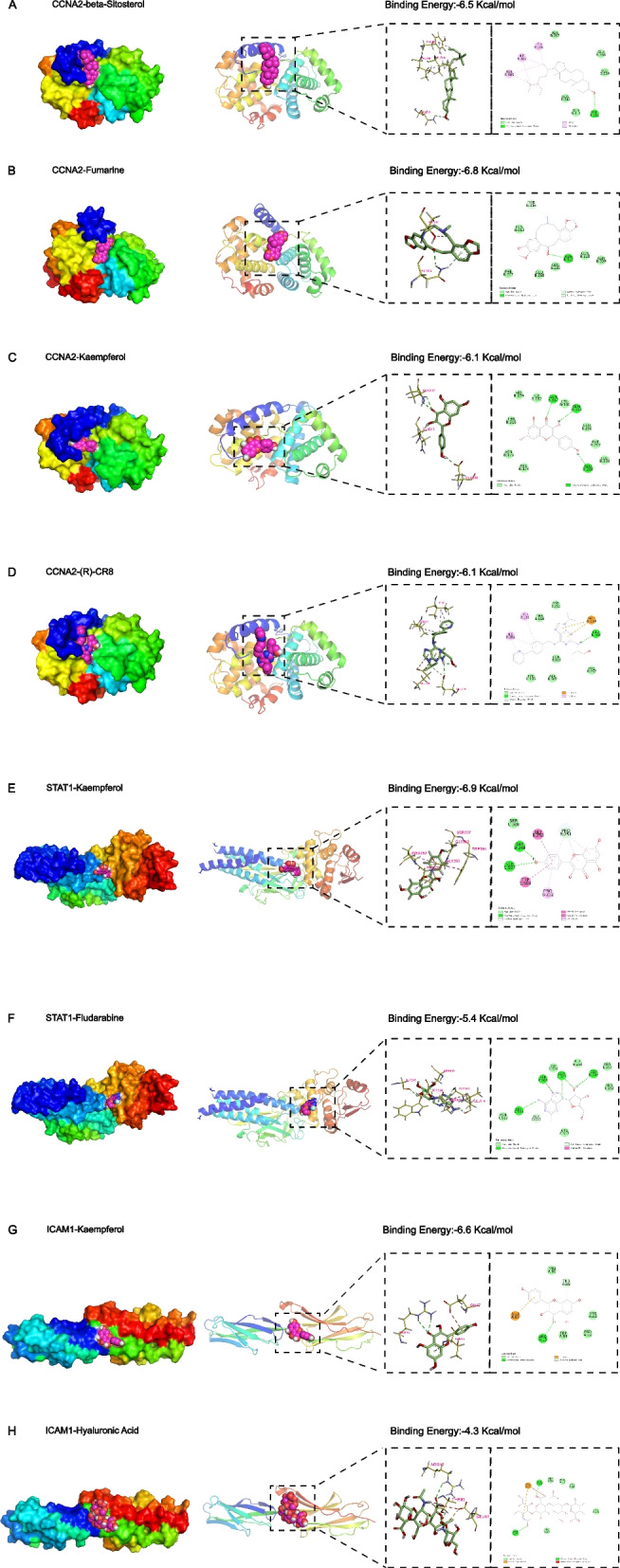
Molecular docking results of core targets and active components

Initially, the molecular docking technique was validated by re-docking the co-crystal ligand into the corresponding site of protein. Additionally, the RMSD value of STAT1 and ICAM1 proteins were found to be 1.634 Å and 0.682 Å (both < 2 Å), respectively, indicating good performance of the employed docking technique. After being validated, the CCNA2, STAT1, and ICAM1 key targets were subjected to molecular docking with associated core ingredients. The molecular docking results were shown in Fig. 5.

For the CCNA2-beta-Sitosterol complex, the binding energy was − 6.5 kcal/mol, and the beta-Sitosterol compound was observed to interact with ASN312 residue through a conventional H-bond. In addition, ILE182 and LEU186 residues formed alkyl bonds with 2 atoms of beta-Sitosterol, while TYR185 residue projected pi-alky bonds with 2 atoms of the ligand, and the remaining five residues interact with the ligand via van der Waals bonds (Fig. 5A).

The CCNA2-Fumarine complex, which had a binding energy of − 6.8 kcal/mol, formed a conventional hydrogen bond and a pi-donor hydrogen bond through the interaction of Fumarine with ASN312 residue. Furthermore, THR316 residue interacted with the ligand via a carbon hydrogen bond, and van der Waals bonds were also involved in residue-ligand interactions (Fig. 5B).

In addition, a binding energy of − 6.1 kcal/mol between Kaempferol and CCNA2 was revealed, and the GLU268, GLN313, and GLN317 residues were suggested to interact with Kaempferol via three conventional hydrogen bonds. Besides hydrogen bonds, Kaempferol also formed several van der Waals bonds with other residues (Fig. 5C).

While (R)-CR8, as a reference inhibitor of the CCNA2 protein, had a binding energy of − 6.1 kcal/mol and formed a conventional hydrogen bond with GLU268 residue, two pi-anion bonds with GLU230 residue, a carbon hydrogen bond with GLU268, and three pi-alkyl bonds with ILE182 and LEU186 residues. The remaining residues formed interactions with the ligand via van der Waals bonds (Fig. 5D).

The binding energy of the STAT1-Kaempferol complex was − 6.9 kcal/mol. Kaempferol formed two conventional hydrogen bonds through interactions with GLY249 and SER507 residues. Other than that, GLY250 and TRP504 residues interacted with Kaempferol through amide-pi stacked and pi-pi T-shaped bonds, respectively. PRO251 residue as well as PRO252 residue were involved in the formation of pi-alkyl bonds by interacting with the Kaempferol compound. Additionally, the PRO251 residue interacted with the Kaempferol compound through a carbon hydrogen bond, whereas the SER508 residue engaged with the Kaempferol via a van der Waals bond (Fig. 5E).

STAT1-Fludarabine complex was − 5.4 kcal/mol. Fludarabine interacted with GLY249, SER503, SER507, and LEU514 residues through five conventional hydrogen bonds, and interacted with SER503 and GLY513 residues through an amide-pi stacked bond and a pi-donor hydrogen bond, respectively. Additionally, six additional residues were observed to interact with Fludarabine through van der Waals forces (Fig. 5F).

The binding energy of the ICAM1-Kaempferol complex was − 6.6 kcal/mol, and the ARG13 residue was found to interact with the Kaempferol compound, which was linked by a conventional hydrogen bond. It was observed that the GLU87 and THR85 residues interacted with kaempferol through a pi-anion and a pi-donor hydrogen bond, respectively. The remaining residues interacted with the ligand via van der Waals bonds (Fig. 5G).

The binding energy of the ICAM1-Hyaluronic acid complex was − 4.3 kcal/mol, and Hyaluronic acid interacted with THR85 and ARG13 residues on the target protein by two hydrogen bonds. GLU87 residue formed an attractive charge by interaction with the ligand. In the vicinity of Hyaluronic acid, five residues were observed to engage in interaction through van der Waals bonds (Fig. 5H).

### Cellular Thermal Shift Assay (CETSA)

**Fig. 6:**
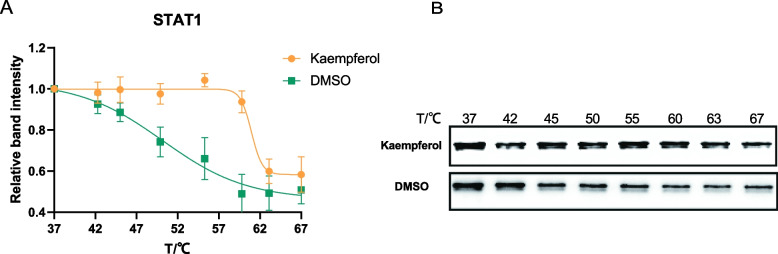
CETSA for interaction between Kaempferol and STAT1. **A** Line chart statistical results. **B** Results of western blot

To validate whether Kaempferol can bind directly to the STAT1 target, CETSA experiments were conducted. Compared to the DMSO group, the melting curve of Kaempferol was significantly shifted to the right, and the Tm value was changed from 50.18 ℃ to 60.96 ℃ after dosing treatment. In addition, the thermal stability of the STAT1 protein was significantly higher than that in the control group under the intervention of kaempferol, indicating that Kaempferol had a binding effect with STAT1 (Fig. 6).

### Molecular dynamics simulation

**Fig. 7:**
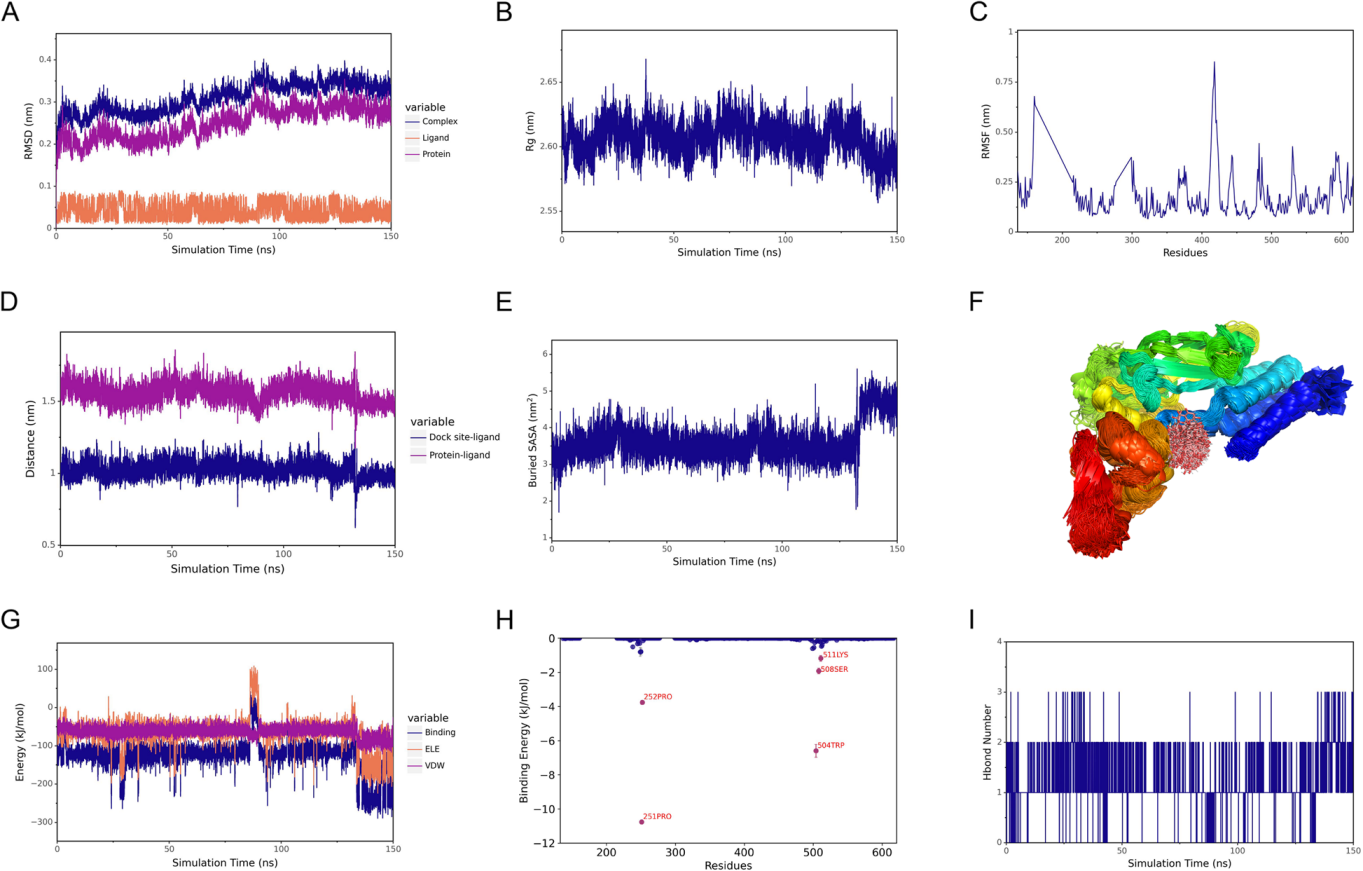
MD simulation analysis of STAT1-Kaempferol complex for 150 ns. **A** The RMSD plot of complex, protein, and small molecule ligand. **B** The Rg plot of STAT1-Kaempferol complex. **C** RMSF plot of the protein. **D** Spacing of protein-small molecule binding sites. **E** Buried SASA. **F** Modelling conformational superposition. **G** Analysis of electrostatic and van der Waals force interactions (ELE indicates electrostatic interaction, VDW indicates van der Waals forces and hydrophobic interactions, and binding indicates the sum of VDW and ELE.). **H** Amino acid binding energy contribution. **I** Hbond number. RMSD, root mean square deviation; RMSF, root mean square fluctuation; Rg, radius of gyration; SASA, solvent-accessible surface area

Based on the molecular docking results, 150 ns molecular dynamics simulations were executed on the STAT1-Kaempferol complex. As depicted in Fig. 7A, the RMSD curves for both the STAT1-Kaempferol complex and the STAT1 protein showed a slight rise in the initial stage of the simulation and remained stable after 100 ns. From the analysis of the Rg plot in Fig. 7B, it can be seen that the Rg value of the ligand–protein complex exhibited few alterations, suggesting that the overall conformation of the proteins remained in a stable status. The RMSF plot in Fig. 7C demonstrated that the fluctuation in amino acid residues with the STAT1 protein ranged from 0.1 to 0.8 nm. Notably, a lower RMSF value was revealed in the region where amino acids were bound to or close to small molecules, which indicated stable binding between protein and ligand. As shown in Fig. 7D, the distances between the small molecule and the protein center, as well as between the small molecule and the initial binding site, exhibited minimal fluctuations and remained consistently stable. Buried SASA reflected the size of the binding interface where small molecules bound to proteins. As shown in Fig. 7E, buried SASA essentially stayed constant and was increased noticeably between 135 and 150 ns, indicating a further enhancement of molecule-protein binding. In addition, the simulated trajectory was processed to superimpose the simulated conformation, and the result was shown in Fig. 7F. There was a high degree of small-molecule stacking, and the small molecule was always bonded to the initial site. According to Fig. 7G, the Van der Waals (VDW) curve exhibited the smallest fluctuation, while both the VDW and electrostatic (ELE) curves remain stable. Furthermore, Fig. 7H exhibited the energy contribution of amino acid binding. It revealed that the crucial amino acids responsible for binding small molecules in the protein were PRO-251 and TRP-504. Subsequently, in Fig. 7I, the number of hydrogen bonds between the Kaempferol molecule and STAT1 protein mainly fluctuated between 1 and 2.

### MM-PBSA binding free energy calculation

Based on the MMPBSA method, the free energies of binding for proposed complexes were obtained. As shown in Table 2, the binding free energy of Kaempferol-STAT1 complex was − 41.648 ± 2.711 kcal/mol. For the complex, Van der Waals, electrostatic interaction and hydrophobic interaction were the major contributors to the free energies of binding. Among these, Van der Waals force was the dominant interactions, while electrostatic force played a secondary role, and hydrophobic interactions was served as a supporting factor. However, the polar solvation was considered as a negative factor for the generation of the free energies of binding.

**Table 2:** MMPBSA and non-bonded interaction analysis(kcal/mol)

Complex	ΔE_vdw_	ΔE_ele_	ΔE_pol_	ΔE_nonpol_	ΔG_bind_
STAT1-Kaempferol	-93.805 ± 1.677	-64.971 ± 4.051	129.861 ± 5.14	-12.733 ± 0.123	-41.648 ± 2.711

ΔE_vdw_, Van der Waals; ΔE_ele,_ Electrostatic interaction; ΔE_pol,_ Polar Solvation; ΔE_nonpol,_ Non-Polar Solvation

## Discussion

Given the high prevalence, AIC refers to a form of drug-induced cardiomyopathy characterized by symptoms such as irregular heart rhythms and heart failure. The underlying mechanisms of AIC remain incompletely understood, although research has implicated several contributing factors, including oxidative stress, inflammation, mitochondrial dysfunction, intracellular calcium overload, and cellular apoptosis [22]. Current treatment options for AIC are limited, and novel approaches are needed to address this issue. Although previous studies have suggested the potential of ginseng in treating adriamycin-induced cardiomyopathy, the exact mechanism is not fully elucidated. Network pharmacology, integrating data mining and modern systems biology techniques, provides an effective approach to systematically explore the potential mechanisms of traditional Chinese medicine in treating AIC. Therefore, the present study utilized network pharmacology to explore the active compounds, potential targets and signaling pathways of ginseng in the treatment of AIC, and further investigated the binding modes and affinities between the active compounds and potential targets through molecular docking, CETSA, and molecular dynamics simulations. In the present study, we employed network pharmacology techniques to examine the potential targets and mechanisms of ginseng in counteracting AIC. As a result, we constructed a ginseng-ingredients-targets-AIC network, identifying three key ingredients (beta-Sitosterol, Fumarine and Kaempferol) and three key targets (CCNA2, STAT1 and ICAM1). Enrichment analysis revealed that ginseng might exert protective effects against AIC via multiple molecular pathways, including AGE-RAGE in diabetic complications, fluid shear stress and atherosclerosis, and TNF signaling pathway. Furthermore, molecular docking, CETSA and MD simulation confirmed the strong binding affinity and structural stability between the three bioactive compounds and the three core target proteins, providing additional support for the potential efficacy of ginseng in mitigating AIC. These findings comprehensively reveal the potential therapeutic targets for ginseng against AIC and provide theoretical bases for the future drug development of ginseng.

Numerous studies have highlighted the outstanding position of some traditional Chinese medicine (TCM) formulas in the treatment of AIC via multiple mechanisms and ginseng usually act as the monarch's cure in most of these formulas [23, 24]. For example, the classical TCM formula Shenmai injection was demonstrated to alleviate adriamycin-induced myocardial injury by regulating the expression of miR-30a/Beclin 1 and activating the Nrf2/Keap1 signaling pathway [25, 26]. Similarly, the Danshen dripping pill can exert cardioprotective effects by inhibiting DOX-regulated expression of molecules involved in oxidative stress, cardiac fibrosis, apoptosis, inflammation, and energy metabolism in the heart [27]. Moreover, in the DOX-induced H9C2 cardiomyocyte injury model, Wenyang Zhenshuai granules could promote ERK5 protein expression and phosphorylation by regulating LncRNA-MiR143HG/miR-143, thereby playing a protective role in the heart [28]. In addition, several significant monomers ginseng glycoproteins and ginsenosides in ginseng have also shown anti-inflammatory, anti-oxidant, and anti-apoptotic action on AIC by previous studies, further supporting the cardio-protection role of ginseng [10–12]. However, given the diversity of active compounds and targets in TCMs, it is difficult to elucidate the precise mechanism of ginseng extracts in the treatment of AIC, while the studies employing ginseng monomers to investigate the cardio-protective effect is powerless to comprehensively showcase the mechanism of ginseng. Therefore, the present study employed network pharmacology, molecular docking, and molecular dynamics to comprehensively explore the potential mechanisms of action of ginseng in the treatment of AIC.

Twenty-two ingredients were screened according to our criterion, among which three ingredients including Kaempferol, beta-Sitosterol, and Fumarine with a greater abundance of potential therapeutic targets were deemed as key ingredients for ginseng action against AIC. In both in vivo and in vitro experiments, Kaempferol has displayed promising effects in attenuating AIC by modulating various cellular pathways, including P53 function, ERK1/2 activation, ROS reduction, and improvement of mitochondrial function [29, 30]. Beta-Sitosterol is one of the components of the ginseng extract, exhibiting anti-inflammatory, antioxidant, anticancer, and lipid-lowering multiple biological functions [31]. Beta-Sitosterol was found to reduce H9C2 apoptosis and oxidative damage in rat cardiomyocytes by upregulating the cellular glutathione redox cycle [32]. Furthermore, research conducted by Lin et al. demonstrated that beta-Sitosterol could inhibit apoptosis and reduce oxidative stress by modulating PPARγ/NF-κB signaling, which in turn reduced apoptotic area and myocardial infarction in rats [33]. Considering the known properties of AIC, the present study implied that beta-Sitosterol-induced inhibition of apoptosis and oxidative stress may be potential mechanism accounting for the treatment of AIC. However, the exact molecular mechanism of beta-Sitosterol against AIC is unclear and remains to be further investigated. Fumarine, also known as protopine, is a ginseng extract that belongs to a class of isoquinoline alkaloids. Fumarine has garnered attention for its diverse pharmacological properties [34]. Previous studies have shown that in carrageenan (CA)-induced mice model and LPS-induced BV2 cells, Fumarine inhibited the expression of iNOS and COX-2 genes and decreased the secretion of inflammatory factors through the NF-κB and MAPK pathways, thereby exerting anti-inflammatory properties [35]. Along with that, Fumarine also exerted antioxidant effects by inhibiting intracellular Ca2 + influx, caspase-3 activation and cell apoptosis [36]. Moreover, an experimental study demonstrated that Fumarine reduced the incidence of arrhythmias in a variety of animal models of experimental arrhythmias [37]. Although the present study holds the promise that Fumarine might be a potential candidate to treat AIC, studies are needed to ascertain the specific molecular mechanism. Whether Fumarine is acting as antioxidant and/or anti-inflammatory modulator needs to be further verified.

In this study, CCNA2, STAT1 and ICAM1 were identified as crucial targets of ginseng in the treatment of AIC. CCNA2 is a crucial regulator of cell proliferation, its sustained expression in the heart invokes cardiomyocyte mitosis to promote cardiomyocyte proliferation and regeneration [38], thereby improving cardiac function in rodents with myocardial infarction and heart failure [39, 40]. The present study employing differential expression analysis revealed a significant down-regulation of CCNA2 subjected to DOX treatment in hiPSC-CMs. These findings suggested that CCNA2 might be a potential therapeutic target to counteract AIC. STAT1, a gene that is implicated in signal transduction and transcriptional activation, plays critical roles in regulating cell apoptosis and fate [41]. STAT1 is extensively expressed in immune cells, endothelial cells, and cardiac myocytes in the heart and is an important modulator of cardiac injury [42]. Previous studies showed that STAT1 expression was increased in DOX-induced rat cardiomyocytes, and inhibition of STAT1 gene expression or JAK2/STAT1 signaling pathway reduced DOX-induced ferroptosis and myocardial fibrosis in rat cardiomyocytes, thereby alleviating heart failure [43, 44]. Consistent with previous findings, the transcriptome analysis in the present study also revealed the increased expression of STAT1 in hiPSC-CMs with DOX treatment. These findings collectively suggested that targeting STAT1 might serve as a potential therapeutic approach to alleviate AIC. ICAM1 is an immunoglobulin-like adhesion factor which is mainly expressed in leukocytes and endothelial cells, and has a variety of biological activities, including the regulation of leukocyte trafficking, participation in the immune response and inflammatory response [45]. Previous studies have demonstrated that ICAM1 is a key factor mediating pathological cardiac remodeling and heart failure [46]. Elevated levels of ICAM1 have been associated with cardiovascular diseases such as atherosclerosis and heart failure [47]. Similarly, ICAM1 expression was found to be increased in rat models with DOX treatment [48], which was in accordance with the finding in the present study, suggesting a possible correlation between increased ICAM1 expression and AIC.

The enrichment analysis results indicated that the molecular signaling mechanisms underlying the protective effect of ginseng against AIC primarily involved AGE-RAGE in diabetic complications pathway, Fluid shear stress and atherosclerosis, and TNF signaling pathway. In a rodent model of AIC, DOX was found to cause cardiomyopathy by promoting the formation of pentosidine and AGEs, which was associated with increased ROS production [49]. Notably, blocking the interaction between AGEs and RAGE was shown to inhibit the activation of cardiac fibroblasts and ameliorate cardiac dysfunction in mice [50], suggesting the significance of the AGEs-RAGE signaling pathway in AIC. Shear stress responses to friction generated by blood flow on the endothelium surface of the vessel wall plays an important role in the regulation of vascular endothelial homeostasis. Noteworthily, endothelial dysfunction is a significant factor that results in the cardiotoxic effects of DOX [51]. Moreover, it was found that fluid shear stress activated p53 and the pro-apoptotic protein BAX by increasing the generation of reactive oxygen species, contributing to DOX-induced endothelial toxicity [52]. Interestingly, fluid shear stress-induced vascular endothelial injury and subsequent atherosclerosis was ever found to be ameliorated by ginsenoside Rg1 by modulating the FAK-PI3K/Akt signaling pathway [53]. These finding suggested that ginseng might be able to improve cardiovascular endothelial function and attenuate adriamycin-induced cardiac injury by interfering with fluid shear stress and atherosclerosis signal pathway. The strong correlation between AIC and cell apoptosis mediated by inflammatory factors was evidenced by previous studies [22]. TNF-α is a significant pro-inflammatory cytokine that can trigger myocardial cell apoptosis signaling and exacerbate myocardial inflammatory necrosis in both acute and chronic cardiac inflammation [54]. Activation of TNF-α signaling was revealed by many previous studies, while inhibition of TNF-α signaling provided cardioprotective role in in-vitro and in-vivo models of AIC [55, 56].

The docking results demonstrated that the binding energies of all the complexes, except for the STAT1-Hyaluronic acid complex, were less than − 5.0 kcal/mol, indicating strong binding capabilities between the core active ingredients and targets. Among the compounds which bound to the CCNA2 protein, the shortlisted compounds have lower binding energies as compared to the reference drug (R)-CR8, indicating higher bonding abilities of the compounds towards the CCNA2 protein. Similarly, screened ingredients had favorable binding potential with STAT1 and ICAM1 proteins in comparison to the reference inhibitors. In these evaluations, STAT1-Kaempferol complex was found to have the strongest bonding ability. Kaempferol was a compound with polar groups and aromatic rings that could form hydrogen bonds and hydrophobic interactions with residues of the STAT1 protein due to its unique structural features. These interaction bonds increased the binding affinity of Kaempferol to STAT1 protein. Additionally, the CETSA experiment verified the interaction between protein and ligand, and molecular dynamics simulation further confirmed the good bonding ability and stability of STAT1-Kaempferol complex. This result supported the potential cardioprotective effect of ginseng on AIC involving effector compounds beta-Sitosterol, Fumarine, and Kaempferol by targeting CCNA2, STAT1, and ICAM1.This investigation possessed several restrictions, which must be acknowledged. Initially, the methodology employed in network pharmacology depends heavily on preexisting databases for both data analysis and network construction. Regrettably, the anticipated drug constituents and objectives might exhibit variability due to the autonomous nature of data and sizable disparities in data incorporation amongst diverse databases. Therefore, verification of the accuracy and comprehensiveness of the data was needed. Furthermore, the present network pharmacology analysis examined solely the interconnection between elements and targets; thus, it failed to offer evidence regarding the precise dose–response association. Lastly, despite the successful identification of core targets and signaling pathways, additional validation via fundamental research and clinical experiments is necessary to establish the mechanisms definitively.

Nevertheless, the strength of this study lies in its inaugural utilization of a bioinformatics approach that integrates network pharmacology, molecular docking, and molecular dynamics, enabling a comprehensive and systematic investigation of the mechanism of action of ginseng in mitigating AIC. To date, few studies have comprehensively explored the mechanism of ginseng treatment of AIC. In this study, a variety of bioinformatics methods were comprehensively used, which overcame the defects of previous research to some extent. In addition, the present study also proposed several future prospects with regard to the potential concurrent use of ginseng with pharmacotherapy. The specific role and mechanisms of action in treating different types of tumors remain unclear and warrant further investigation, although ginseng has been demonstrated to possess anti-tumor properties in colon cancer, breast cancer, lung cancer and gastric cancer. [57]. Then, despite previous research indicated that ginseng was low or even nontoxic in vivo [7], further investigation is required to ascertain its safety in patients with tumors. Moreover, the effect of ginseng on the safety and efficacy derived from anti-tumor therapy is an unresolved issue, and their in-depth mechanisms and coping strategies need to be further studied in the future.

## Conclusion

In summary, the present study elucidated the potential mechanisms of ginseng against AIC with network pharmacology, suggesting that ginseng might exhibit cardioprotective effect through multiple compounds, targets, and pathways. CCNA2, STAT1, and ICAM1 proteins were the most promising targets for ginseng in the treatment of AIC through effector compounds beta-Sitosterol, Fumarine and Kaempferol. The cardioprotective action of ginseng mainly involved AGE-RAGE in diabetic complications, fluid shear stress, and atherosclerotic and TNF signaling pathways. Although our findings provided valuable insights into the potential mechanisms of ginseng in treating AIC, further experimental validation was necessary to substantiate the findings of this study.

## Supplementary Information

Supplementary Material 1.

## Data Availability

The data that support the findings of this study are available from the corresponding author.

## Abbreviations

DOX: Doxorubicin
AIC: Anthracycline-induced cardiotoxicity
ROS: Oxygen species
DEX: Dexrazoxane
CETSA: Cellular thermal shift assay
MD: Molecular dynamics simulations
TCMSP: Traditional Chinese medicine systems pharmacology
OB: Oral bioavailability
DL: Drug-likeness
ETCM: Encyclopedia of traditional Chinese medicine
hiPSC-CMs: Human induced pluripotent stem cell-induced cardiomyocytes
GEO: Gene Expression Omnibus
DEGs: Differentially expressed genes
PPI: Protein-protein interaction
DC: Degree centrality
BC: Betweenness centrality
CC: Closeness centrality
EC: Eigenvector centrality
LAC: Local average connectivity-based method
NC: Network centrality
GO: Gene ontology
KEGG: Kyoto encyclopedia of genes and genomes
BP: Biological process
MF: Molecular function
RMSD: Root mean square deviation
CCNA2: Cyclin A2
STAT1: Signal transducer and activator of transcription 1
ICAM1: Intercellular adhesion molecule 1
(R)-CR8: (2R)-2-({9-(1-methylethyl)-6-[(4-pyridin-2-ylbenzyl)amino]-9H-purin-2-yl}amino)butan-1-ol
RESP: Restrained electrostatic potential
LINCS: Linear constraint solver
PME: Particle mesh ewald
NVT: Canonical ensemble
NPT: Constant-pressure, constant-temperature
RMSF: Root mean square fluctuation
Gg: Radius of gyration
SASA: Solvent accessible surface area
MMPBSA: Molecular Mechanics Poisson-Boltzmann Surface Area
VDW: Van der Waals
ELE: Electrostatic interaction
TCM: Traditional Chinese medicine

## Data availability

No datasets were generated or analysed during the current study.
