# Supplementary material for: Exploring the molecular mechanism of ginseng against anthracycline-induced cardiotoxicity based on network pharmacology, molecular docking and molecular dynamics simulation
Source: Hereditas. 2024 Sep 6;161:31. doi: 10.1186/s41065-024-00334-y (PMC11378563; doi:10.1186/s41065-024-00334-y)
Supplement: Supplementary file 1 — Supplementary Material 1. [file 41065_2024_334_MOESM1_ESM.docx]

Table S1. Active ingredients and effective targets of Ginseng

| Active Ingredients | Effective Targets |
| --- | --- |
| Aposiopolamine | DPP4、CHRM3、ADRB2、CHRM1、DPP4、SLC6A3、SLC6A2、SLC6A4、GABRA1 |
| Arachidonate | ABCA1、ALDH2、ALDH3A1、ALOX5、CCND1、C1R、CASP3、CDK2、CDK4、CETP、COL1A2、EGF、G6PD、GLB1、KCNK2、NOS2、NOS3、PECAM1、PLA2G4A、PPARG、PPP5C、PRKCB、MAPK1、PTEN、PTGS1、PTGS2、RELA、SELP、TNFRSF1A、TNFRSF1、UCP2、PTGES、ABCG1、ABCC4、HERC5、KCNK10、RETN、PTGES2、NCOA2、RXRG、CA2、ACHE |
| beta-Sitosterol | PTGS2 、HSP90AB1、HSP90AA1、PIK3CG、KCNH2 、DRD1 、SCN5A 、GABRA2、PDE3A 、HTR2A 、GABRA5、ADRA1A、BAX 、AKR1C1、AKR1C2、CLEC4E、GABRA3、GABRA4、GABRA6、GABRB1、GABRB2、GABRB3、GABRD、GABRE、GABRG1、GABRG2、GABRG3、GABRP、GABRQ、GRIN1、GRIN2A、GRIN2B、GRIN2C、GRIN2D、GRIN3A、GRIN3B、HSD17B1、LIP3、LSS、NR1I2、NR1I3、PPARA、RORA、SIGMAR1、SULT2A1、SULT2B1、VDR、BAX、BCL2、CASP3、CASP8、CASP9、JUN、MAP2、PON1、PRKCA、TGFB1、ACHE、CHRM4、DRD1、NR3C1、CHRM1、NCOA2、DPP4、CHRNA2、CHRNA7、GSK3B、CCNA2、SCN5A、CHRM3、PDE3A、PGR、CDK2、CA2、ESR1、PPARG、PRSS1、ADRB2、AR、NOS2、PTGS1、PIM1、DPEP1、CHEK1、GABRA1、SLC6A4、PTGS2、NR3C2、OPRM1、MAPK14、ADRA1B、KCNH2、CHRM2、ADRA1D、ESR2 |
| Deoxyharringtonine | AR、NR3C2 |
| Dianthramine | HSP90AA1、HSP90AB1、PTGS1、CDK2、PTGS2、ESR1 |
| Diop | ADRB2 、ADRB2、SCN5A、CHRM3、PRSS1、DPP4、ESR1、PPARG |
| Frutinone A | F2、DPP4 、HSP90AA1、HSP90AB1、PIK3CG、SLPI 、RXRA、NOS2、PDE3A、CDK2、PPARG、GABRA1、PTGS2、CCNA2、GSK3B、ESR2、CA2、AR、ACHE、DPP4、MAPK14、CHRNA7、PTGS1、ADRB2、ESR1、CHEK1、SCN5A、PIM1 |
| Fumarine | HTR2A、HSP90AA1、HSP90AB1、CALM1、CALM2、TOP2A、TOP2B、ADRB2、CALM3、CHRM1、HTR3A、KCNH2、KDR、OPRD1、OPRM1、PTGS1、PTGS2、SCN5A、SLC6A3、SLC6A4、CHEK1、ACHE、DPP4、CHRM4、PIM1、ADRA1B、MAPK14、CACNA1S、PPARG、CHRM3、PDE3A、CDK2、CCNA2、AR、GSK3B、F7、ADRA1D、ESR2、CHRM5、PRSS1、ESR1、DRD1、NOS2 |
| Ginsenoside rh2 | NOS2、GAB1、KDR、FASN、BAX 、PTGS2、PSMG1、MAP2K4、SLC2A4、ANXA1、ATP1A1、ATP1A2、ATP1A3、GABRB3、GLRA3、HSD11B1、HSD11B2、HSD3B1、IL6、LPL、NFKB1、NFKB2、NR3C1、PPP1CC、SLCO1B3、TNF、VDR、YWHAE、FAS、CASP8、CASP9、TNFRSF1A、TP53、BAK1、BCL2、ADCYAP1、BAX、CASP1、CASP3、IFNG、IL1B、NFKBIA |
| Ginsenoside-Rh4_qt | NR3C2、NR3C1、ESR1、AR、NCOA2 |
| Girinimbin | PIK3CG、ADRB2、PTGS1、PTGS2、RXRA、SCN5A、PRSS1、CDK2、CCNA2、ESR2、CHEK1、ACHE、PIM1、MAPK14、ESR1、AR、GSK3B、GABRA1、CHRNA7、NCOA2、DPP4 |
| Gomisin B | TOP2A 、TUBA4A 、TUBB、ESR2、ACHE、NCOA2、ESR1、PTGS2、AR、PRSS1、PIM1、PDE5A |
| Inermin | PTGS2 、SLC6A4 、HSP90AA1 、HSP90AB1 、PIK3CG、CALM1、CALM2、ADRB2、CALM3、HTR3A、IGHG1、PRSS1、PTGS1、PTGS2、RXRA、SCN5A、SLC6A4、NCOA1、CA2、ADRA1D、CHEK1、CDK2、CCNA2、ESR2、CHRNA7、CHRM3、GSK3B、PIM1、NOS2、AR、ESR1、DPP4、MAPK14、ADRA1B |
| Kaempferol | NOS2 、PTGS1、AR、PTGS2、HSP90AB1、DPP4 、PRSS1、PGR、F2、CHRM1、NOS3、GABRA2、SLC6A2、ADRA1B、GABRA1、TOP2B、F7、CALM1、CALM2、RELA、IKBKB、BCL2、BAX、TNF、JUN、AHSA1、CASP3、MAPK8、XDH、MMP1、STAT1、CDC42、PPARG、HMOX1、CYP3A4、CYP1A2、CYP1A1、ICAM1、SELE、VCAM1、NR1I2 、CYP1B1、ALOX5、HAS2、GSTP1、PSMD3、SLC2A4、NR1I3、INSR、DIO1、PPP3CA、GSTM1 、GSTM2、AKR1C3、SLPI 、ACTB、AHR、AKR1C1、AKT1、ATP5A1、ATP5B、ATP5C1、CA1、CA12、CA14、CA3、CA4、CA5A、CA5B、CA6、CA7、CA9、CBR1、CDK6、CEBPB、COMT、CSNK2A1、CSNK2B、CYP19A1、DHFRL1、DNMT1、EIF3F、ESR1、ESR2、ESRRA、ESRRB、GABRA3、GABRA4、GABRA5、GABRA6、GABRG1、GABRG2、GABRG3、GPER1、HCK、HIBCH、HSP90AA1、HSPA2、IGHG1、JAK1、KANSL3、MTTP、NCOA1、NCOA2、NQO2、NR1I2、PIK3CG、PIM1、PRKACA、PRKCA、PRKCB、PTK2B、RUVBL2、SF3B3、SHBG、SOAT1、SOAT2、SQLE、STK17B、SYK、TOP2A、UBA1、UGT3A1、ACHE、CALM3、DIO1、GSTM1、NOS2、POLD1、SLPI、MAPK14、CDK1、DPEP1、CDK2、GSK3B、CHRM2、CCNA2、CA2、CHEK1、DPP4 |
| Malkangunin | ESR1 |
| Panaxadiol | ESR2、NR3C1、MMP9、AR |
| Stigmasterol | PGR、NR3C2、NCOA2、ADH1C、IGHG1、RXRA、NCOA1、PTGS1、PTGS2、ADRA2A、SLC6A2、SLC6A3、ADRB2、AKR1B1、PLAU、LTA4H、MAOB、MAOA、CTRB1、CHRM3、CHRM1、ADRB1、SCN5A、HTR2A、ADRA1A、CHRM2、ADRA1B、CHRNA7、AKR1C1、AKR1C2、AR、CLEC4E、ESR1、ESR2、GABRA1、GABRA2、GABRA3、GABRA4、GABRA5、GABRA6、GABRB1、GABRB2、GABRB3、GABRD、GABRE、GABRG1、GABRG2、GABRG3、GABRP、GABRQ、GRIN1、GRIN2A、GRIN2B、GRIN2C、GRIN2D、GRIN3A、GRIN3B、HSD17B1、LIP3、LSS、NR1I2、NR1I3、NR3C1、PPARA、RORA、SIGMAR1、SULT2A1、SULT2B1、VDR、BACE1、APP、ABCC2、ABCG5、ABCG8、ABCB11、NR1H4、NR1H3、NR1H2、TLR4、ABCA1、HMGCR、SLCO1B1、PRSS1、CDK2、DPEP1、ADRA1D、PPARG、CA2、NOS2、DPP4、ACHE |
| Suchilactone | HSP90AA1、HSP90AB1、KCNMA1、CALM1、CALM2、ADRB2、CALM3、KCNH2、PTGS1、PTGS2、RXRA、SCN5A、NCOA1、PDE3A、MAPK14、ESR1、PPARG、NOS2、DPP4、PIM1、F7、ADRA1D、CHEK1、GSK3B、AR、ESR2、CDK2、PRSS1、CCNA2 |
| Alexandrin_qt | PGR |
